# Listening to Puns Elicits the Co-Activation of Alternative Homophone Meanings during Language Production

**DOI:** 10.1371/journal.pone.0130853

**Published:** 2015-06-26

**Authors:** Sebastian Benjamin Rose, Katharina Spalek, Rasha Abdel Rahman

**Affiliations:** 1 Institut für Psychologie, Humboldt-Universität zu Berlin, Berlin, Germany; 2 Institut für deutsche Sprache und Linguistik, Humboldt-Universität zu Berlin, Berlin, Germany; University of Hyderabad, INDIA

## Abstract

Recent evidence suggests that lexical-semantic activation spread during language production can be dynamically shaped by contextual factors. In this study we investigated whether semantic processing modes can also affect lexical-semantic activation during word production. Specifically, we tested whether the processing of linguistic ambiguities, presented in the form of puns, has an influence on the co-activation of unrelated meanings of homophones in a subsequent language production task. In a picture-word interference paradigm with word distractors that were semantically related or unrelated to the non-depicted meanings of homophones we found facilitation induced by related words only when participants listened to puns before object naming, but not when they heard jokes with unambiguous linguistic stimuli. This finding suggests that a semantic processing mode of ambiguity perception can induce the co-activation of alternative homophone meanings during speech planning.

## Introduction

Speaking, even in the case of producing single words, involves the activation of multi-faceted meaning components at the conceptual and lexical level, and with identical words different aspects of meaning can be conveyed. For instance, depending on the speaker’s intentions and the conversational context, the verbal description “blue” may be meant metaphorically, rather than literally [1], the remark “fantastic” may be used to express enthusiasm or, as an ironic statement, the opposite state of mind [2], and “downhill” may be an intentionally ambivalent description when talking about the development of one’s skiing skills. Thus, everyday language often conveys ambiguities and multi-layered meaning. Yet, research on the production of utterances with multiple alternative meanings is scant, and very little is known about effects of broader semantic and conversational contexts on word production.

The present study was designed to investigate such complex co-activations during the production of homonyms. Homonyms are words that are pronounced or spelled the same way (e.g., ball) but differ in their meanings (sport device vs. gathering for a dance). Precisely, words that are spelled the same way but differ in meaning are called homographs while words that are pronounced the same way but differ in meaning are called homophones, and homonyms are the superordinate term encompassing both. For the purpose of this study, however, we are using the terms homophone and homonym synonymously. Moreover, we do not distinguish between homonyms and polysemes. Polysemes, in contrast to homonyms, are ambiguous words that are related by a shared semantic origin; however, in practice, the distinction is often difficult to make.

**Fig 1:**
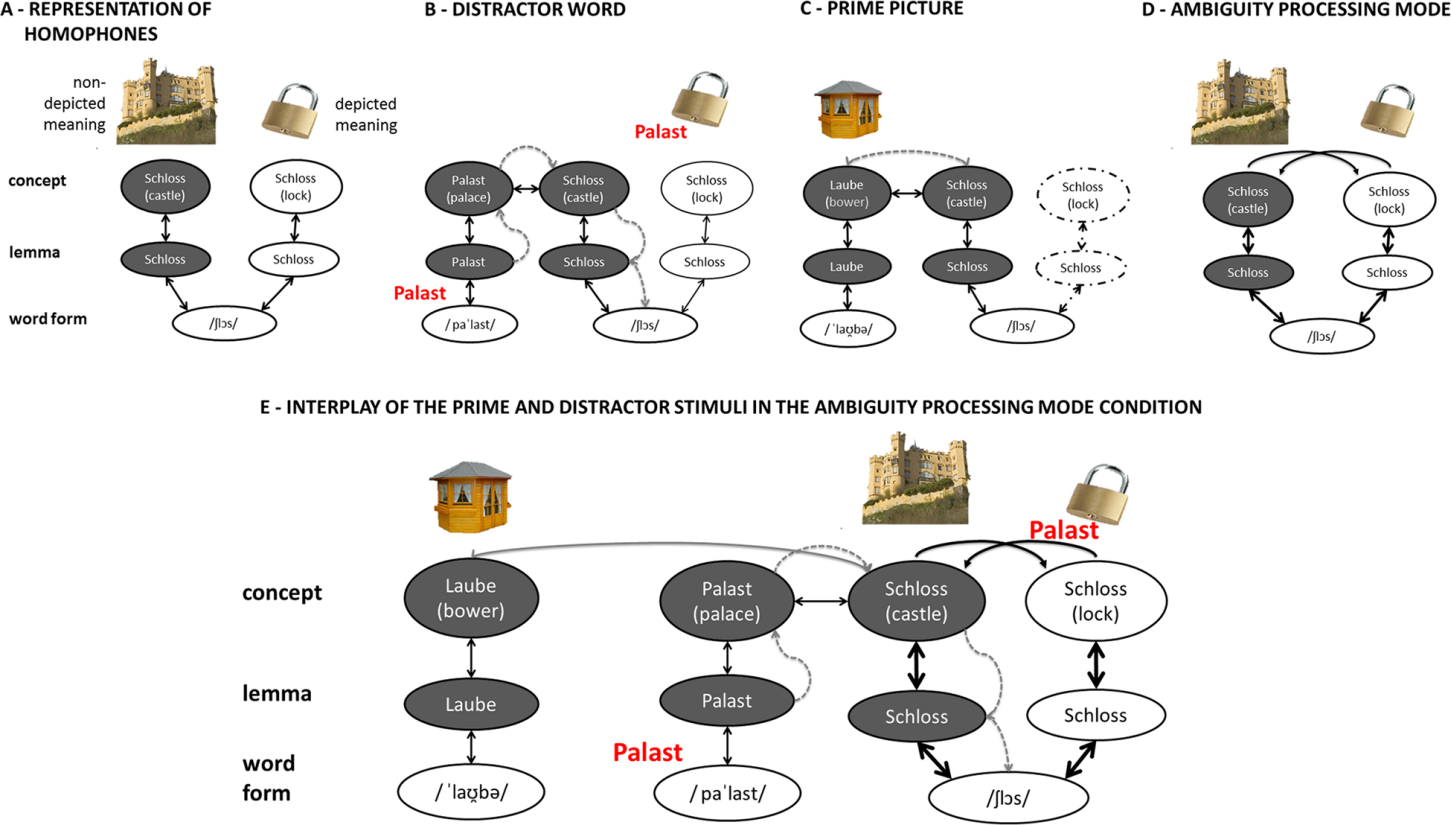
Models explaining effects of prime relatedness, distractor relatedness and ambiguity processing mode on naming of pictures with homophone names. (A) Representation of homophones: homophones have different conceptual and lexical representations but share the same word form. (B) Cascading information flow of distractor words related to the non-depicted meaning of the homophone and pre-activation of the shared word form. (C) Pre-activation of the non-depicted meaning by a previously presented prime stimulus that is categorically related to the non-depicted meaning of the target‘s name. (D) Listening to puns leads to the co-activation of both meanings of a homophone due to enhanced feedback between phonological, lexical and semantic stages and thus the ambiguity status of a target‘s name is quickly available. (E) Interplay of the prime and distractor stimuli in the ambiguity processing mode condition.

Within speech production models homophones are assumed to share the same phonological code but have different conceptual and lexical representations [3, 4–9] (see Fig 1A). Here, we test whether alternative meanings of homophones are co-activated during word production when the speaker was recently exposed to ambiguous verbal messages in the form of puns. Thus, we ask whether the production system can be biased towards co-activating alternative meanings during homophone production by providing a context of ambiguity processing.

A priori, the co-activation of the alternative—and semantically unrelated—meanings of homophones in a picture naming experiment is rather unlikely. However, as the existence of numerous puns demonstrates, we are able to generate such ambiguous utterances in every-day life, and we seemingly enjoy the simultaneous activation of two meaning alternatives when producing or listening to puns [10]. Accordingly, the language system seems to be well-equipped for representing ambiguities, and this holds for the production as well as for the comprehension side.

Furthermore, ample evidence suggests that the way in which language is produced may be shaped by prior experiences. For instance, studies on structural priming have demonstrated a tendency to produce sentences with the same syntactic form if that form has recently been employed or processed during comprehension [11–13]. Such findings on structural priming from comprehension to production have been taken as evidence for shared mechanisms between language comprehension and production. Moreover, several semantic priming studies have shown that processing of subliminally presented stimuli depends on the attentional configuration of the semantic system by task relevant information (gating framework; for a review see [14]). Therefore, task relevant or contextual information modulates the semantic activation spread according to task affordances so that processing of information that is congruent with the task set or context is enhanced. Here we suggest, somewhat analogously, a priming mechanism from perception to production at the semantic level that does not depend on concrete semantic similarities but rather on a biased semantic processing mode (cf. [15]). Specifically, we propose that the confrontation with lexical ambiguities during pun comprehension will induce a conceptual ambiguity processing mode that is transferred to speech production, triggering the co-activation of alternative meanings of homophones. Crucially, context-induced conceptual co-activation of seemingly unrelated meanings has recently been demonstrated in a picture naming study [16], suggesting that the dynamics of semantic activation spread are adapted flexibly by situational contexts. Such effects indicate a considerable level of flexibility at the semantic side of the speech production system.

### Language production and the co-activation of meaning alternatives

According to most models of language production, preparing for speaking involves conceptual, lexical and morpho-phonological processes as basic components. During picture naming, not only the conceptual and lexical representations of the target utterance, but also those of semantically related concepts and lexical representations are activated [17–19]. Because many models assume that conceptual and lexical representations are shared between production and comprehension, the transmission of information between the two levels is assumed to be continuous and bi-directional [17]. Thus, at the lexical level, the appropriate candidate must be selected from among co-activated entries. For instance, when we name a picture of a dog, concepts and lexical entries of related objects such as cat and rabbit are simultaneously activated to some degree. Furthermore, some evidence suggests that co-activated lexical entries pass some activation to their word forms [20–22], resulting in the co-activation of semantically related alternatives of the message at the conceptual, lexical and phonological level.

Evidence for co-activation patterns at different levels of production comes from the picture-word interference (PWI) paradigm in which pictures of objects (e.g., bee) are named while simultaneously presented word distractors should be ignored. When the distractor has a semantic-categorical relation to the target (e.g., ant), naming times are longer compared to the presentation of unrelated words (e.g. [18, 23, 24]). This effect has been interpreted to reflect competition for selection of co-activated entries at the lexical level (but see [25]).

In the case of homophones sharing only the word forms but not meaning aspects, the alternative meanings should not be co-activated on a regular basis in a similar manner as demonstrated for categorical relations. Nevertheless, distractors that are categorically related to the non-depicted meanings of homophones in a PWI task have been shown to facilitate naming relative to unrelated words [5, 26]. This finding is accounted for by the shared word forms of homophones. Distractors that are related to the alternative meaning activate the lexical representation of the alternative and, due to continuous information flow between processing stages, its word form (see Fig 1B). Because the word form is shared by both meaning alternatives, resulting in converging activation at the word form level, phonological encoding of the target word is facilitated. Notably, this account does not assume that the meaning alternative is co-activated at the conceptual level in the course of picture naming. However, as mentioned above, recent findings suggest that lexical-semantic co-activation of seemingly unrelated meanings can be induced by context manipulations. The context manipulation in the form of puns which is assumed to induce such co-activation for homophones is outlined below.

### The present study

**Fig 2:**
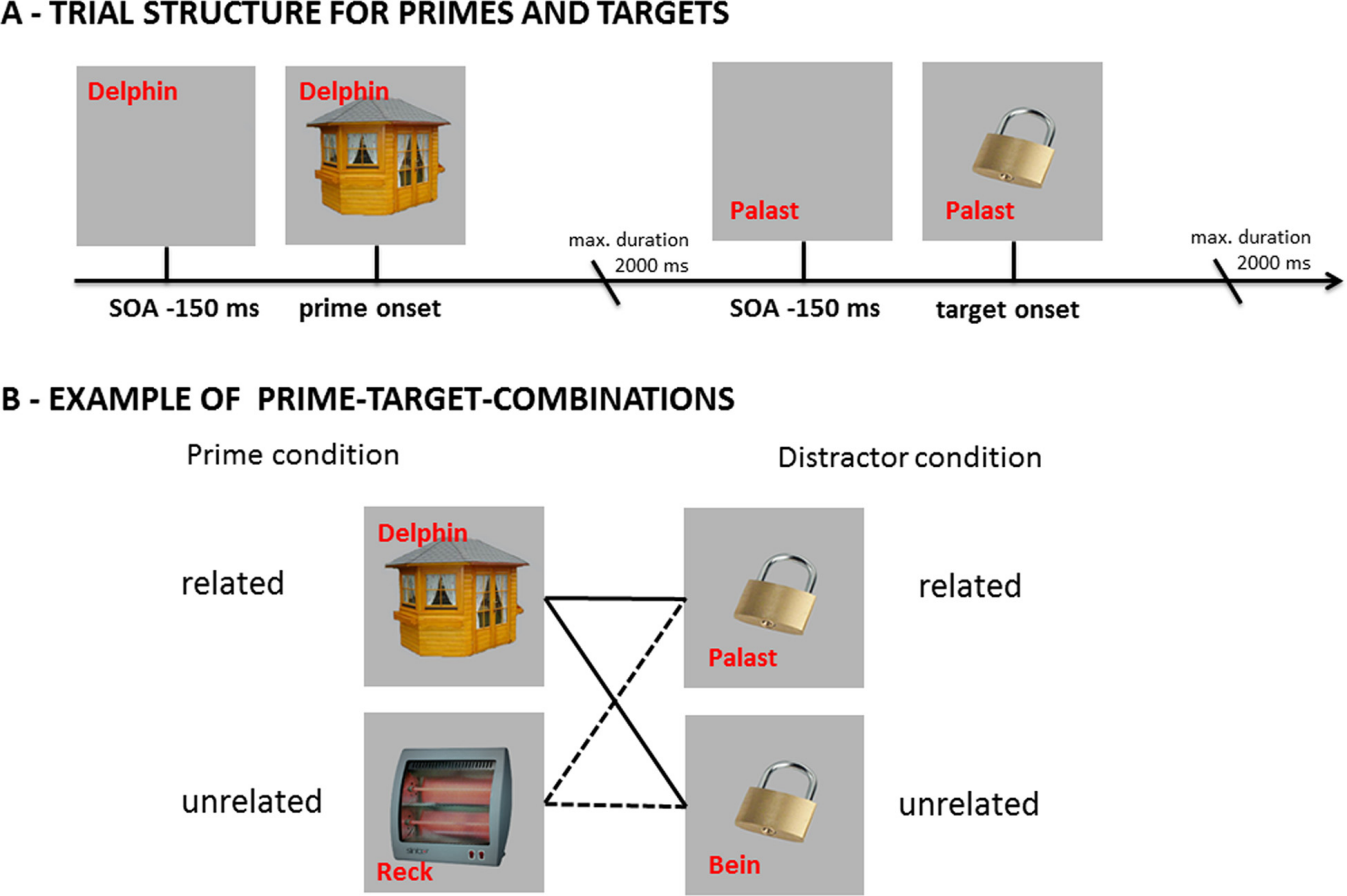
Trial structure for prime and target stimuli and combinations of the prime and distractor conditions. (A) The target picture set consisted of objects with homonymous names. For example: a picture of a lock called “Schloss” (also denoting a castle). Prior to target presentation, a prime stimulus was presented that was categorical related to the non-depicted meaning of the homonymous name. Here, the picture of bower called “Laube” is categorically related to the non-depicted meaning (castle) of the following target. -150 ms before picture presentation a distractor word was presented for the prime and target stimuli. The distractor word was categorically related or unrelated to the non-depicted meanings of target stimuli (e.g. “Palast” (palace) related to the non-depicted meaning of a castle). In the prime condition distractor words were always unrelated. (A) The prime stimulus presented one trial before the targets could be categorically related or unrelated to the non-depicted meaning of the target‘s homophone name and was always unrelated to the depicted meaning. Each prime condition (related: “Laube” (bower), unrelated: “Heizlüfter” (heater)) was crossed with the two distractor conditions of the target stimuli (related: “Palast” (palace); unrelated: “Bein” (leg)).

The goal of this study was to examine whether the planning of ambiguous messages or, more specifically, the co-activation of homophone meaning alternatives, can be elicited by the processing of ambiguities during prior comprehension. To do so, we asked two groups of participants to name pictures of objects with homonymous names in a PWI task, presenting distractor words that were categorically related or unrelated to the non-depicted meanings. For example, for the German homonym “Schloss” (meaning alternatives: lock and castle) we presented a picture of a lock and the distractor word “Palast” (palace; categorically related to non-depicted meaning) or “Bein” (leg; unrelated; see Fig 2 and S1 Table: Used stimuli material). As previously discussed, differences between these distractor conditions can be accounted for on the basis of the shared word form and without assuming concomitant co-activations of the unrelated meanings at the conceptual level during production [5].

Crucially, in order to trigger conceptual co-activations, and thus the production of ambiguous messages, we manipulated the comprehension contexts before and during the naming experiments. One group of participants listened to puns before picture naming and in the breaks between blocks of naming trials. Puns are humorous plays on words in which the punch line depends on two possible and plausible endings in form of a homophone (e.g.: “Two cannibals are eating a clown. One says to the other: ‘It tastes kind of funny.’”; see also S2 Table: Used jokes and puns). The listener has to simultaneously co-activate both meanings of the ambiguous word to understand and appreciate the pun. Thus, puns do not contain one adequate solution, both meanings remain plausible, and they are funny only if the two alternative meanings are co-activated [27, 28]. Therefore, the listener remains in a constant mode of ambiguity processing. Furthermore, in this mode the word form plays a crucial role for semantic processing such that only the shared word form links the two alternative meanings (see Fig 1D).

A fMRI study investigating ambiguity processing during pun comprehension showed that the co-activation of meaning alternatives in puns and their appreciation was correlated with a modulation of activity in the left anterior part of the inferior frontal and temporal gyrus [10]. These regions have been reported to play an important role in speech planning processes (e.g. [29]). Because of shared representations between language comprehension and production, this mode of ambiguity processing may be transferred to the production system. Consequently, a calibration of semantic activation spread and modulated information transmission between phonological, lexical and conceptual planning levels may result in the form of, for instance, enhanced feedback from word form to lexical-semantic processing levels, as outlined below.

As a control condition, the second group of participants listened to jokes. Unlike puns, jokes are characterized by a context or particular storyline and a violation of expected outcomes at the punch line leading to a perceived incongruence (e.g.: “‘Doctor, doctor, when I touch my knee it hurts and when I press on my butt it hurts as well.’–‘Hmm, yes, your finger is broken.’”; see also S2 Table: Used jokes and puns). The comprehension of a joke depends on the listener’s ability to shift the perspective to the storyline, resulting in an adequate solution to resolve the experienced incongruence of the punch line [10, 30–34]. Thus, although participants in both groups were confronted with funny items that may lift their mood or enhance their motivation in a similar way, an ambiguity processing mode was induced only in the puns group, but not in the jokes group.

To summarize, participants in the puns group were in a constant mode of ambiguity processing, induced by repeatedly listening to puns. We hypothesize that this ambiguity processing mode is transferred to the production system. This in turn may result in a calibration of lexical-semantic activation patterns and modulated transmission of information between phonological, lexical and conceptual planning levels (biased automatic information processing, cf.[15]). For instance, because in the ambiguity processing mode during comprehension and production the word forms play a crucial role since they alter semantic processing, the information transmission between the word form and lexical-semantic processing stages (and particularly the feedback activation) may be enhanced (see Fig 1D). Therefore, we expect a differential pattern of co-activations of meaning alternatives of homophones only in the puns but not in the jokes group. This should be reflected in differences between distractors that are related or unrelated to the non-depicted meanings of the homophones exclusively in the puns group.

Prior evidence has demonstrated facilitative effects of distractors that are categorically related to non-depicted meanings of homophones [5, 26]. Therefore, our context manipulation should enhance this facilitative effect by inducing additional (and converging) activation at conceptual, lexical and phonological representations in the course of picture naming. Furthermore, we assume that the interplay between processing levels will be enhanced by feedback connections from phonology (for evidence for such feedback connections between word form and lexical representations, see: [7, 23, 35, 36]). Such a feedback mechanism can explain how conceptual representations of the unrelated meanings of a homophone and their lexical entries that share only phonological information but not semantic information can be simultaneously activated in the course of picture naming.

Finally, taking into account that the traces of the co-activation of semantically unrelated meaning alternatives should be subtle, and given that the effects of phonological co-activation [20–22, 37], and dynamic context adaptations [16] are expected to be weak, we included an additional semantic priming manipulation that should enhance the prospect of finding even subtle effects (see Fig 1C; cf. [38]). To strengthen the conceptual activity of the non-depicted meaning alternatives, we presented pictures of objects with unambiguous names (e.g. “Laube” (bower)) in trials immediately preceding target trials that were categorically related (or unrelated) to the non-depicted alternative meaning of the homophone (e.g. castle; see Fig 2). All prime trials had the same structure as the experimental trials, and the same procedure was realized in both groups of participants.

To summarize, we expected effects (or enhanced effects) of distractors that are categorically related to the non-depicted meaning of a homophone when the homophone had been previously primed. This was expected to occur only in the group that was exposed to ambiguities in the form of puns, and not in the group that was exposed to regular jokes. We therefore predicted a three-way interaction between these experimental factors.

## Methods

### Participants

Eighty-eight participants, aged 18 to 39 years (*M* = 26, *SD* = 4.9), were paid for their participation in the experiment or received partial fulfilment of a curriculum requirement. Participants were randomly assigned to the two groups. There were 17 men and 27 women in the puns group, aged 19 to 38 years (*M* = 26.9, *SD* = 5.4) and 15 men and 29 women in the jokes group, aged 18 to 39 years (*M* = 26.2, *SD* = 4.4). There were no group differences in gender (χ² (1, *N* = 88) = 0.1, *p* > .05) or age (*t*(86) = 0.68, *p* > .05). All participants were native German speakers and reported normal or corrected-to-normal visual accuracy and normal colour vision.

### Ethics Statement

The study was approved by the ethical review board of the Department of Psychology at the Humboldt—Universität zu Berlin in accordance with the Declaration of Helsinki. All participants gave written informed consent prior to their participation in the present study.

### Materials

Target pictures consisted of 60 color photographs of objects with ambiguous names (e.g. lock ("Schloss"); see S1 Table: Used stimuli material). In a pretest, homonymous names were rated according to the dominance of one of their meanings: Seventeen participants who did not take part in the main experiment were presented with a list of 134 ambiguous words. For each of the words, both possible meanings were presented (e.g., “Schloss”: lock/ castle), and participants rated on a seven-point scale how strongly they associated those meanings with the word (1 = “exclusively associated with meaning 1”, 7 = “exclusively associated with meaning 2”). Based on these ratings we selected 20 objects with ambiguous names that had equally dominant meanings (i.e., ratings ranging from 3.5–4.5), and 40 objects with ambiguous names that had only one dominant meaning (i.e., ratings lower than 3.5 or higher than 4.5). For one half of the 40 objects with a dominant meaning we depicted objects showing the dominant meaning and for the other half we presented objects showing the non-dominant meaning. This was done because we expected that the degree of target meaning dominance of the (non-)depicted meaning could influence the impact of the distractor related to the non-depicted meaning (cf.[4, 5, 9]).

Each PWI picture (e.g., the picture of a lock called “Schloss”) was paired with a distractor word that was related (e.g., “Palast” (palace)) or unrelated (e.g. “Bein” (leg)) to the non-depicted meaning of the homophone (e.g., castle; see Fig 2 and S1 Table: Used stimuli material). The unrelated distractor condition was created by re-pairing the words and pictures. In addition to the critical stimuli, we used prime stimuli to enhance the activation levels of the non-depicted alternative meanings. Prime stimuli consisted of 60 color photographs of objects with unambiguous names that were paired with unrelated distractors to assure a similar appearance to the target stimuli (see S1 Table: Used stimuli material and Fig 2). Primes were presented one trial before targets and were related or unrelated to the non-depicted meaning of the target name (see Fig 2). For example, in the related prime condition the picture of a “Laube” (bower) is categorically related to the non-depicted meaning (castle) of the homophone “Schloss” but categorically unrelated to its depicted meaning (lock). By presenting objects with unambiguous names we additionally intended to reduce participants’ potential expectations during the experiment that only homophones would be named. Photographs of target and prime stimuli were scaled to 3.5 cm x 3.5 cm.

Puns and jokes were collected from various sources and recorded by a professional speaker (theatre and TV actor). Afterwards they were rated according to their funniness in a previous study: A total of 75 puns and 75 jokes were distributed across two lists with 75 items each (list A containing 37 puns and 38 jokes; list B containing 38 puns and 37 jokes). Thirty participants who did not participate in the main experiment rated the jokes on a scale from 1–7 (1 = “not funny at all”, 7 = “extremely funny”). Half of the participants responded to list A, the other half to list B. We also asked participants whether they already knew a particular item and whether they found it offensive. To ensure that possible effects in our experiment were not due to different levels of funniness, we selected 25 puns and 25 jokes matched in funniness rating (*M* = 3.41, *SD* = 0.33) for this study (see S2 Table: Used jokes and puns). The puns or jokes did not involve the words that were later included in the experimental prime and target conditions.

### Procedure and Design

Prior to the experiment participants were familiarized with the pictures. Participants were asked to name each picture spontaneously and were corrected by the experimenter if necessary. Subsequently, participants were given a sheet with all objects and their names. The main experiment was performed using Presentation software (Version 0.70, www.neurobs.com). At the beginning of the experiment (and before the PWI task started), each participant listened to fifteen jokes (jokes group) or puns (puns group). The remaining ten jokes or puns were presented in five breaks that subdivided the PWI task to maintain the experimental manipulation throughout the experiment. Jokes and puns (before and in the breaks of the experiment) were randomly presented and participants were asked after listening to each joke or pun to rate the funniness of the presented material on a five-point rating scale (1 = “not funny”, 5 = “very funny”).

Each trial began with a fixation cross in the center of a screen for 0.5 seconds. Then the distractor word written in red color was presented near the target picture. 150 milliseconds after word onset the picture was presented for a maximum of 2 seconds, followed by a blank screen for 1.5 seconds (cf. Fig 2A). The participants’ naming responses were recorded with a voice key. Participants were instructed to name the pictures as quickly and accurately as possible and to ignore the distractors. After naming, the picture disappeared.

Prime stimuli always appeared one trial before the critical PWI pictures and were related or unrelated to the non-depicted meaning of the target (see Fig 2B). Thereby, each prime condition (related, unrelated) was paired with each distractor condition (related, unrelated). Consequently, each picture-word combination was presented twice during the experiment. Additionally, the presentation of prime and PWI stimulus pairs was divided into two parts. In the first part one half of the PWI stimulus set was presented with related primes and the other half with unrelated primes, and vice versa for the second part. Whether a PWI stimulus was presented first with a related or unrelated prime stimulus was randomized for each participant.

### Statistic Design and Analyses

We conducted a linear mixed model (LMM) analysis with crossed random effects for subjects and items (target pictures), including the factors prime relatedness (related or unrelated to the non-depicted meaning of the homophone), distractor relatedness (related or unrelated to the non-depicted meaning of the homophone), target meaning dominance (balanced, dominant or non-dominant) and group (puns or jokes) As reference levels for the prime and distractor relatedness effects we employed the unrelated prime and distractor condition, for the target meaning dominance condition the balanced condition and for the group factor the jokes group (control group). This model structure was mainly driven by our experimental design and hypothesis.

Recently, it has been proposed to include a maximum of by-subject and by-item random slopes because simulations showed that random-intercept only LMMs are anti-conservative and worse than conventional F1/F2 analyses of variance [39]. Random slopes model the source of variance under the factors of interest that is due to variations inside the subject and item sample. We considered several by-item and by-subject random slopes, e.g. by-items slopes for prime relatedness, distractor relatedness and group, and by-subject random slope for prime relatedness. The inclusion of these random slopes did not improve the fit of our original model. However, we did not examine, e.g., a by-subject random slope for group or a by-item random slope. The former would be problematic because different subjects are nested within the level of our group variable (between-subject factor). The latter because there is an insufficient number of observations per unit as in our experiment each item (picture and distractor word) was presented twice but with different preceding primes which represents another source of (error) variance.

Additionally, we also considered the contribution of the covariate visual complexity of pictures to explain additional residual variance because we expected that the effect of our context manipulation, namely the co-activation of unrelated alternative meanings, may be small. However, the portion of explained variance by this covariate was too small to significantly improve the fit of the model.

For the analyses we used R software with the package “lme4” [40]. The reported p-values were derived using the package “languageR” [41] via a Markov chain Monte Carlo (MCMC) simulation.

## Results

### Funniness Ratings

An independent-samples t-test was conducted on the averaged funniness ratings of jokes and puns in the two groups. There was a significant difference between the puns (*M* = 3.6, *SD* = 0.7) and the jokes (*M* = 4.2, *SD* = 0.6) group; *t*(86) = 3.66, *p* < .01. The jokes were perceived as funnier than the puns.

### Naming Latencies

**Fig 3:**
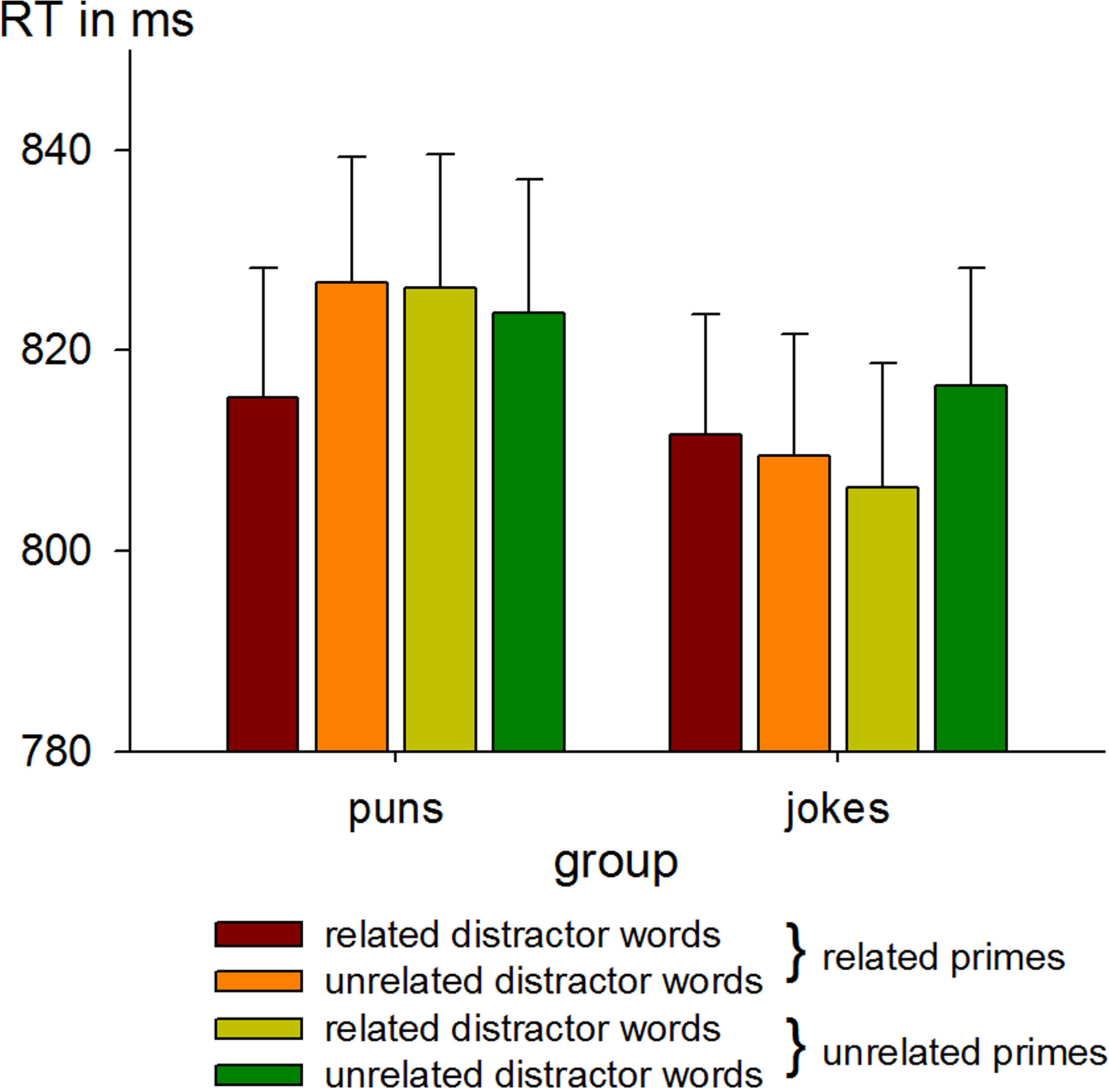
Naming latencies and standard error bars in the puns and jokes group depending on distractor word and prime condition.

Mean RTs in the different conditions are presented in Fig 3 and Table 1. LMM analyses with crossed random effects for subjects and items and the factors prime relatedness, distractor relatedness, target meaning dominance and group revealed a significant interaction between prime relatedness, distractor relatedness and group (β = -43.8, *SE* = 19.3; *t*(20326) = 2.22; *p* < .05), reflecting facilitation for related distractors and primes in the puns group (see Table 2; correlation matrix see S3 Table: Correlations of fixed effects of the overall LMM). For the factors prime relatedness, distractor relatedness, group and target meaning dominance, no main effect occurred, even though, descriptively, a difference of overall RT latency between both groups (puns = 823 ms vs. jokes = 810 ms) and a difference for target meaning dominance in form of a decrease in RTs from the non-dominant (839 ms), to balanced (818 ms) to dominant targets (792 ms) was observed.

**Table 1:** Mean naming latencies (RTs, in milliseconds), standard errors of means (SEs), and standard deviations (SDs) for the prime and distractor word condition separated for both groups.

conditions		puns				jokes			
prime	distractor	RT	SE	SD	Diff [Rel-Unrel]	RT	SE	SD	Diff [Rel-Unrel]
related	related	815,32	12,9	85,8	-11,4	811,66	12	80,2	2,1
	unrelated	826,74	12,5	83,2		809,47	12,2	81,2	
unrelated	related	826,19	13,3	88,3	2,4	806,28	12,5	83	-10,1
	unrelated	823,74	13,3	88,7		816,46	11,7	77,7	

**Table 2:** Model summary and effect estimations of the overall LMM with crossed random effects for subjects and items including the factors prime relatedness, distractor relatedness, target meaning dominance and group.

Model structure:				
RT ~ group * distractor * prime * meaning dominance + (1 | participant) + (1 | item)				
information criterion	AIC	BIC	logLik	deviance
	274317.9	274531.7	274531.7	274263.9
**Random effects**	**Variance**	**Standard Deviation**		
participant (intercept)	6303	79.39		
item (intercept)	6060	77.85		
residual	41291	203.20		
**Fixed effects**	**Estimate**	**Std. Error**	**t value**	**p(>|t|)**
(Intercept)	816.15	22.25	36.69	0.00**
group	5.98	19.60	0.31	0.76
distractor	-8.87	9.85	-0.90	0.37
prime	-3.21	9.84	-0.33	0.74
meaning dominance[nd]	36.60	26.52	1.38	0.17
meaning dominance[d]	-30.65	26.51	-1.16	0.25
group*distractor	14.15	13.97	1.01	0.31
group*prime	13.37	13.97	0.96	0.34
distractor*prime	20.62	13.93	1.48	0.14
group*meaning dominance[nd]	-10.85	14.00	-0.78	0.44
group*meaning dominance[d]	14.24	13.96	1.02	0.31
distractor*meaning dominance[nd]	-13.65	13.95	-0.98	0.33
distractor*meaning dominance[d]	7.31	13.93	0.52	0.60
prime*meaning dominance[nd]	-21.46	13.95	-1.54	0.12
prime*meaning dominance[d]	5.77	13.93	0.41	0.68
group*distractor*prime	-43.87	19.75	-2.22	0.03*
group*distractor*meaning dominance[nd]	7.69	19.78	0.39	0.70
group*distractor*meaning dominance[d]	-12.14	19.73	-0.62	0.54
group*prime*meaning dominance[nd]	13.08	19.78	0.66	0.51
group*prime*meaning dominance[d]	-20.83	19.74	-1.06	0.29
distractor*prime*meaning dominance[nd]	4.95	19.73	0.25	0.80
distractor*prime*meaning dominance[d]	-26.08	19.72	-1.32	0.19
group*distractor*prime*meaning dominance[nd]	3.22	27.95	0.12	0.91
group*distractor*prime*meaning dominance[d]	50.50	27.92	1.81	0.07'

[nd] = non-dominant meaning; [d] = dominant meaning

Subsequent analyses for each prime condition revealed a significant group and distractor relatedness interaction only for related primes (β = -29.8, *SE* = 14.05, *t*(10164) = 2.11; *p* < .05), but not for unrelated primes (β = +14.2; *SE* = 13.8; *t*(10156) = 1.0; *p* > .05; see Table 3, correlation matrix S4 Table: Correlations of fixed effects of the subsequent LMMs separated for the related and unrelated prime condition).

**Table 3:** Model summary and effect estimations of the subsequent LMMs separated for the related and unrelated prime condition.

Model structure: RT ~ group * distractor * meaning dominance + (1 | participant) + (1 | item)								
	Related prime condition				Unrelated prime condition			
information criterion	AIC	BIC	logLik	deviance	AIC	BIC	logLik	deviance
	137495.09	137603.49	-68732.54	137465.09	137184.19	137292.58	-68577.09	137154.19
**Random effects**	**Variance**	**Standard Deviation**			**Variance**	**Standard Deviation**		
participant (intercept)	6126	78.27			6368	79.80		
item (intercept)	5485	74.06			6715	81.95		
residual	41752	204.33			40855	202.13		
**Fixed effects**	**Estimate**	**Std. Error**	**t value**	**p(>|t|)**	**Estimate**	**Std. Error**	**t value**	**p(>|t|)**
(Intercept)	812.908	21.498	37.81	0***	816.014	22.991	35.49	0***
group	19.120	19.416	0.98	0.3264	6.123	19.650	0.31	0.756
distractor	11.804	9.903	1.19	0.2333	-8.628	9.798	-0.88	0.379
meaning dominance[nd]	14.980	25.436	0.59	0.5576	36.843	27.707	1.33	0.188
meaning dominance[d]	-25.515	25.429	-1.00	0.3189	-30.360	27.704	-1.10	0.277
group*distractor	-29.619	14.046	-2.11	0.0350*	14.012	13.896	1.01	0.313
group*meaning dominance[nd]	2.400	14.050	0.17	0.8644	-11.076	13.926	-0.80	0.426
group*meaning dominance[d]	-5.985	14.041	-0.43	0.6699	14.250	13.885	1.03	0.305
distractor*meaning dominance[nd]	-8.709	14.033	-0.62	0.5349	-13.893	13.881	-1.00	0.317
distractor*meaning dominance[d]	-18.579	14.033	-1.32	0.1856	7.142	13.860	0.52	0.606
group*distractor*meaning dominance[nd]	10.915	19.857	0.55	0.5826	7.827	19.680	0.40	0.691
group*distractor*meaning dominance[d]	37.728	19.865	1.90	0.0576	-12.220	19.626	-0.62	0.534

[nd] = non-dominant meaning; [d] = dominant meaning

Planned comparisons for the distractor condition linked with prior presentation of related primes revealed a marginally significant distractor effect in the puns group (*t*(10165) = 1.9, *SE* = 5.5, *p* = .054) and no effect in the jokes group (*t*(10165) = 0.4, *SE* = 5.5, *p* > .05). This confirmed the facilitative effect for related distractor words in combination with related primes in the puns group.

As indicated in Fig 3, there was also an unexpected numerical difference between related and unrelated distractors presented after unrelated primes in the jokes group. Although this effect did not reach statistical significance (see above) we cannot exclude the possibility that the three-way-interaction of the factors prime relatedness, distractor relatedness and group was caused (also) by this difference. Therefore, we examined the reliability of this numerical difference in the jokes group by employing a non-parametric bootstrap approach. We resampled the data 2000 times and fitted our LMM for each bootstrap sample. For each sample that showed a significant three-way-interaction of the factors distractor relatedness, prime relatedness and group we tested whether this interaction could be explained by the effect of distractors in the related prime condition in the puns group or by the effect of distractors in the unrelated prime condition in the jokes group. More precisely, if the three-way-interaction could also be caused by distractor effects in the unrelated prime condition in the jokes group, the number of the bootstrap samples showing a significant effect of distractor relatedness in combination with unrelated primes should be equal to the number of samples revealing a significant effect of distractor relatedness in combination with related primes.

**Fig 4:**
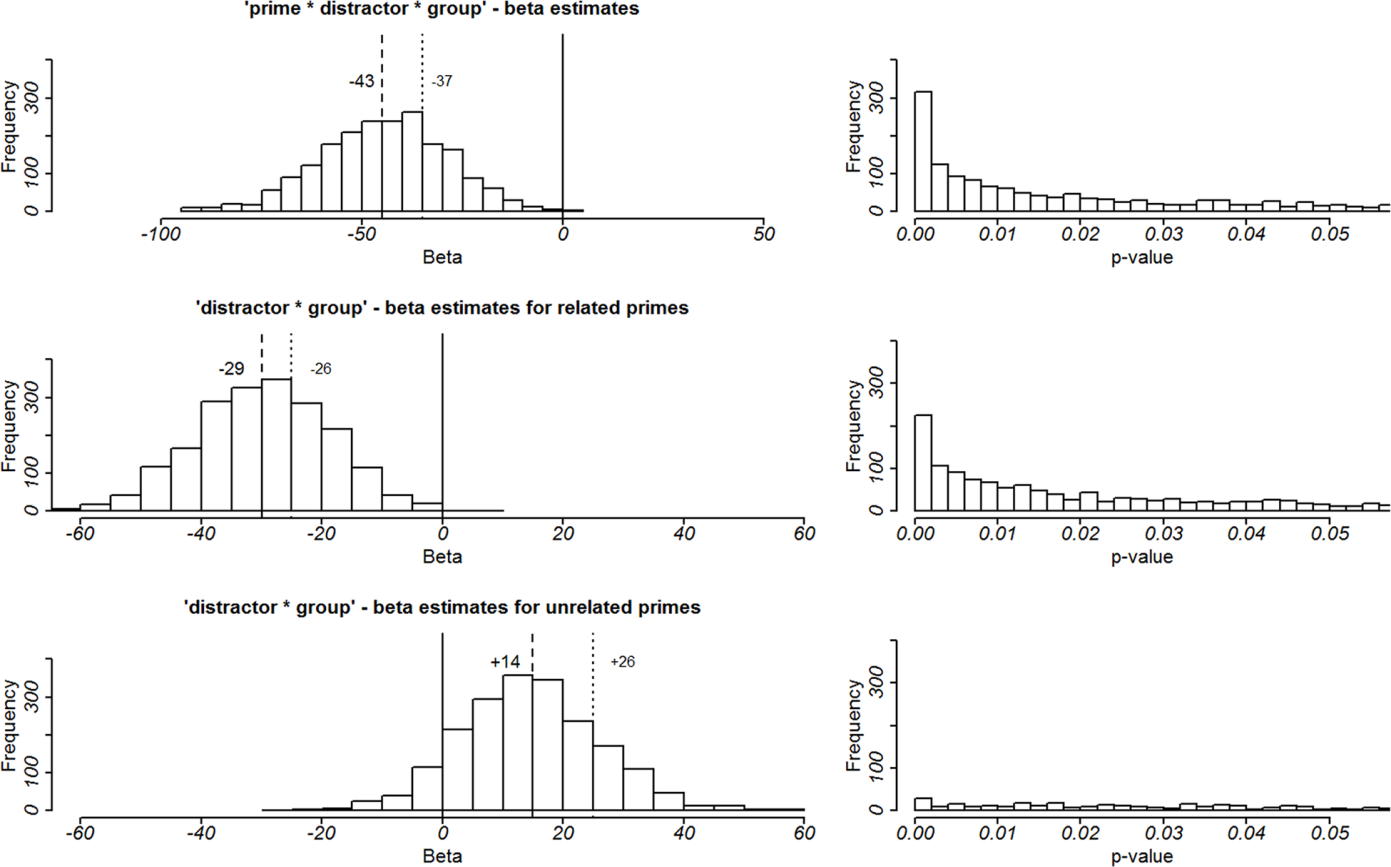
Results of the bootstrap approach: frequency distributions of beta estimates and p-values. Plots on the left show the frequency distribution of beta estimates for the bootstrap samples for the interaction between prime relatedness, distractor relatedness and group (top line), the interaction between distractor relatedness and group in the related (middle line) and unrelated (bottom line) prime condition. The dashed lines show the beta estimate of our original sample. The dotted lines show the minimal value the beta value has to reach to become statistically significant. Plots on the right show the frequency distribution of p-values below 0.05.

First, we determined the frequency distribution representing the resampled means of the beta coefficient for the interaction between prime relatedness, distractor relatedness and group (Fig 4, first line). 63.9% of beta values are distributed below the critical value of—37 that has to be reached to identify the three-way-interaction as statistically significant from zero. This proportion of betas nicely confirms the reliability of the three-way-interaction between the factors distractor relatedness, prime relatedness and group. Second, we selected samples showing a significant three-way-interaction and determined how many of these bootstrap samples showed significant distractor effects in combination with either related or unrelated primes. For both cases the critical value of beta is ± 26.5. The second and third rows of Fig 4 show that 75.14% of samples in the related prime condition exceed this value (in contrast to 21.97% of sample in the unrelated prime condition). Thus, we can conclude that the numerical difference in RTs for unrelated primes in the jokes group is not reliable and therefore will not be discussed any further.

**Fig 5:**
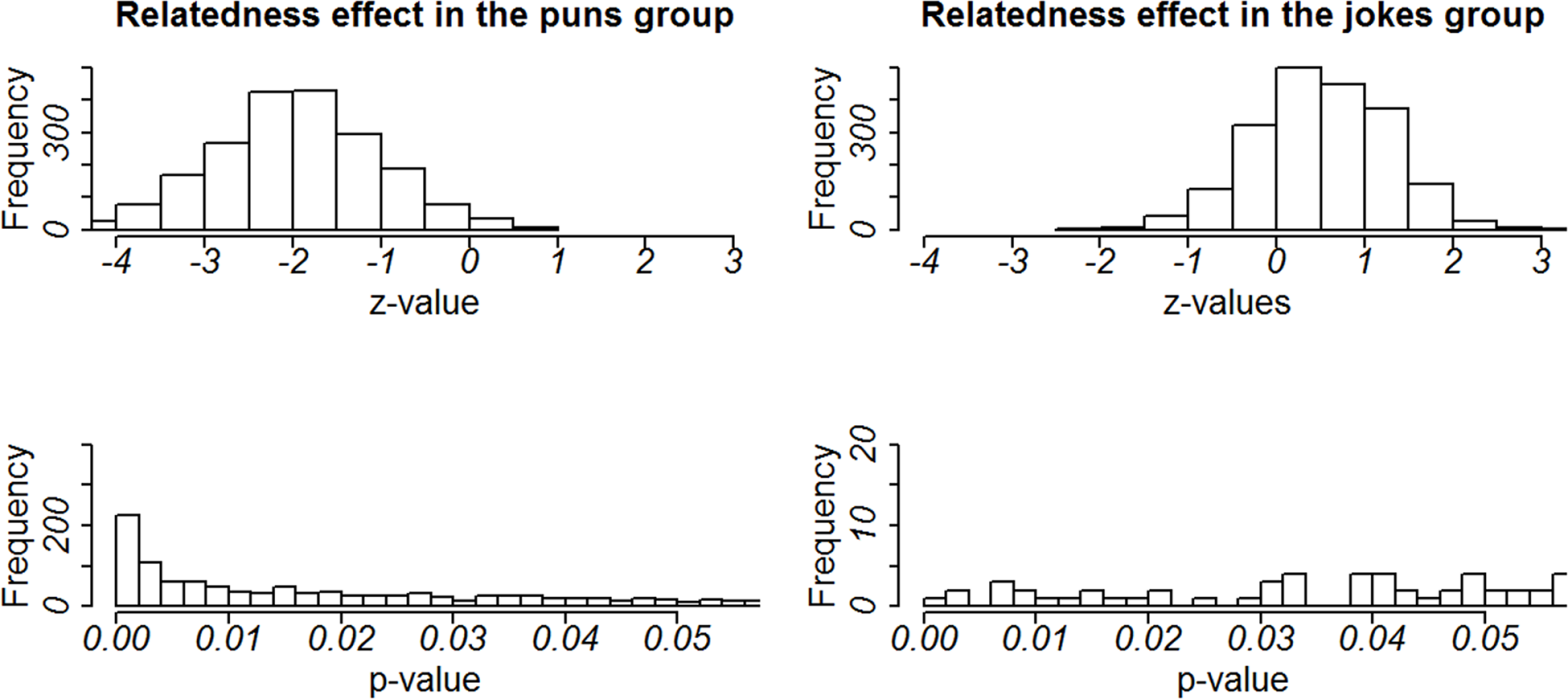
Results of the bootstrap approach: frequency distributions of z-values and p-values for the planned comparisons between related and unrelated distractors in the puns and jokes group.

Furthermore, for samples showing a significant distractor and group interaction in combination with related primes we conducted planned comparisons to investigate whether the distractor relatedness effect in the puns group was also reliable, because this effect was only marginally significant in our main analysis (see above). Fig 5 shows the frequency distribution of z- and p-values for planned comparisons between related and unrelated distractors in the puns and jokes group when related primes were presented before. These distributions nicely confirm that the relatedness effect in the puns group can be reliably found in 65.87% of bootstrapped samples. By contrast a relatedness effect in the jokes group can only be found in 3.42% of samples.

## Discussion

In this study we investigated whether lexical-semantic activation during single word production can be modulated by broader linguistic contexts. Specifically, we tested whether the processing of linguistic ambiguities, presented in the form of puns, can trigger the co-activation of unrelated meanings of homophones during subsequent language production. In a PWI task with word distractors that were semantically related or unrelated to the non-depicted meanings of homophones we found facilitation induced by related words only when the alternative meanings were pre-activated by semantically related prime stimuli in the trials before [38]. As expected (see above), this suggests that the effects are small and subtle [5, 21, 37]. In fact, the additional priming procedure was introduced to enhance the activation levels of the non-depicted alternative meanings because this activation and the effects of distractors that are related to the alternative meanings were expected to be small (see Fig 1C). One could argue that the priming of the categorically related concepts of the non-depicted meaning may have caused (response) priming of the target without the assumption of co-activation of meaning alternatives during picture naming. However, if this were the case, an effect of related primes would also be predicted in the jokes group, which was not the case, as discussed in detail below.

Crucially, facilitation induced by distractors related to the alternative meanings (in primed trials) was observed only when participants were exposed to puns before and in the breaks of the object naming task, but not in the group of participants listening to jokes. This finding can be taken as evidence against the idea that the facilitation is due to direct semantic priming not only of the alternative, but also of the target. It rather shows that the pre-activation of the non-depicted meaning alternatives by prime stimuli was necessary to observe effects of semantic-lexical co-activation in the puns group. Accordingly, the observed facilitative effect in the puns group, and not the jokes group, suggests that a cognitive mode of ambiguity processing during perception can be transferred to the production system, calibrating the spread of activation at lexical-semantic levels and between these levels and the word form level, thereby boosting the word form representations of non-depicted and semantically unrelated meaning alternatives of homophones. Specifically, we assume that the context manipulation has caused the co-activation of alternative meanings, and this may have been triggered and enhanced by feedback connections from the word form to lexical-semantic processing levels (see Fig 1D and 1E).

Due to shared conceptual and lexical representations of the perception and production system and the repeated processing of ambiguities during pun comprehension the activity of the semantic system was modulated. Nevertheless, upon naming a pictured lock (“Schloss”), the semantic system does not, by itself, co-activate the alternative meaning (castle) because the ambiguity status of the target’s name cannot be determined at this level. Its status can only be specified on the basis of the shared word form, and phonology is available only later during speech planning. Thus, we suggest a feedback mechanism from the word form to the lexical-semantic levels of processing [6, 23, 36]. Specifically, during processing of the target, activity from the conceptual level spreads to lexical and phonological representations. After the activation of the shared word form, activity is fed back to higher representational levels, activating to some degree the lexical and conceptual representations of the alternative meaning.

In the pun group the pattern of semantic activation spread is modulated due to the comprehension of linguistic ambiguities. We suggest two mechanisms for this. First, semantic activation spread may be modulated according to the ambiguity mode in the sense of an enhanced sensitivity for ambiguous meaning aspects [14]. Second, the spread of activation in the production network may be modulated. This may be realized by enhanced feedback connections due to higher connection weights between processing levels and in particular between the word form and lexical level [42]. Thus, the generally enhanced status of word forms for semantic processing during pun comprehension may be transferred to the production system and likewise enhance the influence of word forms on lexical and conceptual processing (see Fig 1D). Thus, the impact of this feedback mechanism on the conceptual level is enhanced leading to the co-activation of the unrelated meaning during the production of the picture name, especially when the unrelated meaning alternative has already been conceptually pre-activated by previously presented prime stimuli—and possibly also indirectly by the word distractors, as will be discussed below.

As mentioned in the introduction, distractors that have a semantic-categorical relation to the target have inhibitory effects on naming times. This interference effect has been taken as evidence for lexical co-activation and competition during speech planning (e.g. [18, 23, 24]), because it is assumed that activation from processing of the distractor converges on the same lexical representations that are also activated by the processing of the target picture. In line with this, one may argue that we should have observed an inhibitory effect of distractors that are categorically related to the meaning alternative because the alternative becomes a lexical competitor in the puns group. Although this is theoretically conceivable, we consider this scenario unlikely because not all types of semantic relation induce interference. For instance, associatively related distractors (e.g., target: bee; distractor: honey) have been observed to elicit facilitative effects (e.g. [29, 43, 44]). One reason might be that associates that don’t share a common category frame or a significant amount of semantic features tend to co-activate a very limited number of co-related items, which may in turn result in the active competition of only very few lexical entries (in contrast to the co-activation of many active competitors in case of categorical relations). Therefore, lexical competition might be weak when only one or a few competitors are active, as is the case for associates [44, 45]. A similar argument may hold for distractors that are categorically related to the non-depicted meanings of a homophone. Here, the word form but not the meaning is shared, and the meaning of the alternative is only co-activated when the cognitive system is in the ambiguity processing mode. Thus, even though the alternative meaning of the homophone may be active at the lexical level, and may pass to and receive activation from the word form level, the competition induced by one isolated competitor may be negligible and facilitatory word form effects may therefore dominate. Accordingly, we argue that in the puns group lexical co-activation of the meaning alternative facilitates the naming of the target when the distractor is categorically related to the non-depicted alternative meaning.

It is also conceivable that the processing of the distractor word itself is modulated in the ambiguity processing mode. Distractors were presented 150 ms before target onset and could thus already have induced the co-activation of the alternative meaning through enhanced feedback connections even before target presentation. Notably, facilitatory effects of distractors semantically related to the non-depicted meaning of a homophone have already been found in studies without a manipulation of context in the form of puns [5, 26]. Here, they were explained by converging activation at the word form level that facilitated phonological encoding of the target word (see above and Fig 1B) without assuming the co-activation of the alternative meaning at the conceptual level via feedback mechanisms. But, as the information of the (ambiguity) status of a word form is relevant in the puns group, activation induced by the distractor could have spread from the word form to lexical-semantic stages and may therefore preactivate the alternative meaning even before target processing. Moreover, this explanation would also be in line with the finding that distractor effects in the puns group could only be observed in combination with related prime stimuli, since the activity that is fed back from the word form should only be a small fraction of activation induced by the distractor itself (cf. [21]). Analogously to the explanation for distractor words, the processing of the prime stimuli could also been influenced by ambiguity processing. Accordingly, they would not only alter the resting level of the semantic-lexical representation of the meaning alternative, as mentioned above, but also the feedback links from phonological to lexical-semantic stages that play a crucial role during the ambiguity processing mode.

According to studies reporting facilitation for distractor words semantically related to the non-depicted meaning of a homophone without context manipulation [5, 26], we originally expected an enhancement of facilitatory effects in the puns group by the additional ambiguity manipulation, such that facilitation would be present in the jokes group already and significantly stronger in the puns group as the phonological word form is not only activated by the distractor and target themselves but also by the co-activation of the alternative meaning. However, in contrast to this prediction there was no reliable facilitation effect in the jokes group. Although the goal of this study was not—for the main part—to replicate former studies, there are some differences between our study and these studies that could explain the absence of facilitation in the jokes group. Firstly, Cutting and Ferreira [5] (see also[26]) always presented the dominant meaning of a homophone that was taken from association norms and not from dominance ratings that are less confounded with factors like imageability and concreteness [46]. As can be seen in our study, there is a descriptive difference in RTs between non-dominant (839 ms), balanced (818 ms), and dominant items (792 ms), and general dominance effects cannot be excluded. Moreover, the mentioned studies also used distractors that were directly related to the depicted meaning whereas our distractors were exclusively related to the non-depicted alternative meaning. Cutting and Ferreira [5] and Taylor and Burke [26] constructed the unrelated distractors for both conditions by using words from the direct (distractors related to the depicted meaning) and from the indirect (distractors related to the non-depicted meaning) distractor condition. By this, inherent distractor word effects cannot be fully controlled because, for instance, the impact of related distractor words in the indirect condition are compared with distractors words that were used as related distractors in the direct but not in the indirect distractor condition. Finally, the modality of distractors was different. Cutting and Ferreira [5] (see also [26]) used auditory presented distractors, whereas we presented visual words. Until now, it is still debated whether visual and auditory distractors have the same impact on semantic-lexical processing [47]. Moreover, the presentation of auditory distractors complicates the choice of an optimal stimulus onset asynchrony because the presentation is stretched in time relative to written words [48].

We have found slower RTs in the puns group relative to the jokes group. Even though this difference was not significant, it may be difficult to interpret interactions with a group factor when the groups being compared differ in their baseline RTs [49]. For example, several studies have found that poorer performing groups (in terms of RT) show greater absolute differences between conditions than better performing groups (for a review: [50]). Therefore, one may argue that the effect for related distractor words in combination with related primes might not be specific to the effect of an ambiguity processing mode. However, we consider this unlikely because the group related effect of slower RTs should also influence the difference between related and unrelated distractors in the unrelated prime condition. We did not observe such a difference in the puns group. Accordingly, we conclude that the group difference for related distractors and primes reflects the influence of prior ambiguity processing on semantic activation spread during production. Moreover, slower RTs in the puns group might also be interpretable as direct consequence of the ambiguity processing mode since the co-activation of meaning alternatives might alter information processing times. However, as we found no direct statistical evidence for this explanation in this study, we leave this question to further research.

Finally, in contrast to the initial ratings of the materials, we found a difference in funniness ratings between jokes and puns which may limit the interpretation of our results. The experienced funniness of jokes or puns can induce positive emotions, which in turn enhances mood and may change motivational aspects of a task performance [51]. Positive emotional states are known to affect information processing in many ways, e.g., by promoting processing and integration of new information in the memory system or by enhancing the spread of activation to weak associations [52–54]. Participants in a positive mood are more likely to produce unusual associations and exhibit larger priming effects than participants in a negative mood [55]. Thus, facilitatory effects of related distractors and primes may be caused by a general mood effect leading to enhanced semantic activation spread. However, in the present study, jokes were experienced as funnier than puns. Therefore facilitation for related distractors and primes in the puns group cannot be explained by positive emotions that enhance activation of a widespread association network. Thus, we conclude that facilitation for related distractors in the puns group is caused by ambiguity processing, not by enhanced mood.

To summarize, the effect of related distractors and primes being found only in the puns group, but not in the jokes group, suggests that the prior processing of ambiguities in puns during comprehension has an influence on later co-activation of unrelated meaning alternatives during the production of homophone names. This is a first attempt to describe the production of semantically complex one-word messages with multiple meanings, such as ambiguous messages. Such messages are frequently used in every-day language but still await empirical investigations, and clearly, more research is needed to better understand how such complex utterances are produced. Our study presents a first piece of evidence for the activation of ambiguous messages during single word production. Furthermore, we demonstrate that the linguistic context can calibrate semantic activation spread to trigger ambiguity production.

## Supporting Information

S1 Table Used stimuli material. (DOCX)

S2 Table Used jokes and puns. (DOCX)

S3 Table Correlations of fixed effects of the overall LMM. (DOCX)

S4 Table Correlations of fixed effects of the subsequent LMMs separated for the related and unrelated prime condition. (DOCX)

## References

[1] Glucksberg S. The psycholinguistics of metaphor. Trends in Cognitive Sciences. 2003;7(2):92–6. 10.1016/S1364-6613(02)00040-2 . 12584028

[2] Regel S, Coulson S, Gunter TC. The communicative style of a speaker can affect language comprehension? ERP evidence from the comprehension of irony. Brain Research. 2010;1311:121–35. 10.1016/j.brainres.2009.10.077 . 19900421

[3] Jescheniak JD, Levelt WJM. Word-Frequency Effects in Speech Production—Retrieval of Syntactic Information and of Phonological Form. Journal of Experimental Psychology Learning, Memory, and Cognition. 1994;20(4):824–43. 10.1037/0278-7393.20.4.824 .

[4] Jescheniak JD, Meyer AS, Levelt WJM. Specific-word frequency is not all that counts in speech production: Comments on Caramazza, Costa, et al. (2001) and new experimental data. Journal of Experimental Psychology Learning, Memory, and Cognition. 2003;29(3):432–8. 10.1037/0278-7393.29.3.432 . 12776753

[5] Cutting JC, Ferreira VS. Semantic and phonological information flow in the production lexicon. Journal of Experimental Psychology Learning, Memory, and Cognition. 1999;25(2):318–44. . 10.1037//0278-7393.25.2.318 10093205

[6] Ferreira VS, Griffin ZM. Phonological influences on lexical (mis)selection. Psychological Science. 2003;14(1):86–90. Epub 2003/02/05. . 12564760 10.1111/1467-9280.01424

[7] Bonin P, Fayol M. Frequency effects in the written and spoken production of homophonic picture names. European Journal of Cognitive Psychology. 2002;14(3):289–313. 10.1080/09541440143000078 .

[8] Caramazza A, Bi YC, Costa A, Miozzo M. What determines the speed of lexical access: Homophone or specific-word frequency? A reply to Jescheniak et al. (2003). Journal of Experimental Psychology Learning, Memory, and Cognition. 2004;30(1):278–82. 10.1037/0278-7393.30.1.278 . 14736312

[9] Miozzo M, Caramazza A. The representation of homophones: Evidence from the distractor-frequency effect. Journal of Experimental Psychology Learning, Memory, and Cognition. 2005;31(6):1360–71. 10.1037/0278-7393.31.6.1360 . 16393051

[10] Bekinschtein TA, Davis MH, Rodd JM, Owen AM. Why clowns taste funny: the relationship between humor and semantic ambiguity. The Journal of Neuroscience. 2011;31(26):9665–71. Epub 2011/07/01. 10.1523/JNEUROSCI.5058-10.2011 . 21715632 PMC6485420

[11] van Gompel RPG, Pickering MJ, Pearson J, Jacob G. The activation of inappropriate analyses in garden-path sentences: Evidence from structural priming. Journal of Memory and Language. 2006;55(3):335–62. .

[12] Pickering MJ, Ferreira VS. Structural priming: A critical review. Psychological Bulletin. 2008;134(3):427–59. 10.1037/0033-2909.134.3.427 . 18444704 PMC2657366

[13] Chang F, Dell GS, Bock K. Becoming syntactic. Psychological Review. 2006;113(2):234–72. 10.1037/0033-295x.113.2.234 . 16637761

[14] Kiefer M. Executive control over unconscious cognition: attentional sensitization of unconscious information processing. Frontiers in human neuroscience. 2012;6 . 10.3389/fnhum.2012.00061 PMC3311241 22470329

[15] Joubert OR, Fize D, Rousselet GA, Fabre-Thorpe M. Early interference of context congruence on object processing in rapid visual categorization of natural scenes. Journal of Vision. 2008;8(13):11 1–8. Epub 2009/01/17. 10.1167/8.13.11 . 19146341

[16] Abdel Rahman R, Melinger A. The dynamic microstructure of speech production: Semantic interference built on the fly. Journal of experimental psychology Learning, memory, and cognition. 2011 Epub 2010/09/22. 10.1037/a0021208 . 20854006

[17] Levelt WJ, Roelofs A, Meyer AS. A theory of lexical access in speech production. The Behavioral and brain sciences. 1999;22(1):1–38; discussion -75. . 11301520 10.1017/s0140525x99001776

[18] Schriefers H, Meyer AS, Levelt WJM. Exploring the Time Course of Lexical Access in Language Production—Picture-Word Interference Studies. Journal of Memory and Language. 1990;29(1):86–102. 10.1016/0749-596x(90)90011-N .

[19] Caramazza A. How many levels of processing are there in lexical access? Cognitive Neuropsych. 1997;14(1):177–208. .

[20] Jescheniak JD, Schriefers H. Discrete serial versus cascaded processing in lexical access in speech production: Further evidence from the coactivation of near-synonyms. Journal of Experimental Psychology Learning, Memory, and Cognition. 1998;24(5):1256–74. 10.1037//0278-7393.24.5.1256 .

[21] Peterson RR, Savoy P. Lexical selection and phonological encoding during language production: Evidence for cascaded processing. Journal of Experimental Psychology Learning, Memory, and Cognition. 1998;24(3):539–57. 10.1037//0278-7393.24.3.539 .

[22] Abdel Rahman R, Melinger A. Enhanced phonological facilitation and traces of concurrent word form activation in speech production: An object-naming study with multiple distractors. Q J Exp Psychol. 2008;61(9):1410–40. . 10.1080/17470210701560724 19086192

[23] Damian MF, Martin RC. Semantic and phonological codes interact in single word production. Journal of Experimental Psychology Learning, Memory, and Cognition. 1999;25(2):345–61. . 10.1037//0278-7393.25.2.345 10093206

[24] Damian MF, Bowers JS. Locus of semantic interference in picture-word interference tasks. Psychonomic Bulletin & Review. 2003;10(1):111–7. . 12747497 10.3758/bf03196474

[25] Mahon BZ, Costa A, Peterson R, Vargas KA, Caramazza A. Lexical selection is not by competition: A reinterpretation of semantic interference and facilitation effects in the picture-word interference paradigm. Journal of Experimental Psychology: Learning, Memory, and Cognition. 2007;33(3):503–35. . 10.1037/0278-7393.33.3.503 17470003

[26] Taylor JK, Burke DM. Asymmetric aging effects on semantic and phonological processes: Naming in the picture-word interference task. Psychol Aging. 2002;17(4):662–76. 10.1037//0882-7974.17.4.662 . 12507362

[27] Coulson S, Severens E. Hemispheric asymmetry and pun comprehension: When cowboys have sore calves. Brain and Language. 2007;100(2):172–87. 10.1016/j.bandl.2005.08.009 . 16199084

[28] Sheridan H, Reingold EM, Daneman M. Using puns to study contextual influences on lexical ambiguity resolution: Evidence from eye movements. Psychonomic Bulletin & Review. 2009;16(5):875–81. 10.3758/Pbr.16.5.875 . 19815792

[29] de Zubicaray, Hansen S, McMahon KL. Differential processing of thematic and categorical conceptual relations in spoken word production. Journal of experimental psychology General. 2013;142(1):131–42. 10.1037/a0028717 . 22642711

[30] Coulson S, Kutas M. Getting it: human event-related brain response to jokes in good and poor comprehenders. Neuroscience Letters. 2001;316(2):71–4. . 11742718 10.1016/s0304-3940(01)02387-4

[31] Moran JM, Wig GS, Adams RB, Janata P, Kelley WM. Neural correlates of humor detection and appreciation. Neuroimage. 2004;21(3):1055–60. 10.1016/j.neuroimage.2003.10.017 . 15006673

[32] Coulson S, Wu YC. Right hemisphere activation of joke-related information: An event-related brain potential study. Journal of Cognitive Neuroscience. 2005;17(3):494–506. 10.1162/0898929053279568 . 15814008

[33] Bartolo A, Benuzzi F, Nocetti L, Baraldi P, Nichelli P. Humor comprehension and appreciation: An fMRI study. Journal of Cognitive Neuroscience. 2006;18(11):1789–98. 10.1162/jocn.2006.18.11.1789 . 17069470

[34] Marinkovic K, Baldwin S, Courtney MG, Witzel T, Dale AM, Halgren E. Right hemisphere has the last laugh: neural dynamics of joke appreciation. Cognitive Affective & Behavioral Neuroscience. 2011;11(1):113–30. 10.3758/s13415-010-0017-7 . PMC3047694 21264646

[35] Dell GS. Positive Feedback in Hierarchical Connectionist Models—Applications to Language Production. Cognitive Sci. 1985;9(1):3–23. 10.1207/s15516709cog0901_2 .

[36] Burke DM, Locantore JK, Austin AA, Chae B. Cherry pit primes Brad Pitt—Homophone priming effects on young and older adults' production of proper names. Psychological Science. 2004;15(3):164–70. . 15016287 10.1111/j.0956-7976.2004.01503004.x PMC2255560

[37] Dell GS, O'Seaghdha PG. Mediated and convergent lexical priming in language production: a comment on Levelt et al. (1991). Psychological Review. 1991;98(4):604–14. Epub 1991/10/01. . 1961775 10.1037/0033-295x.98.4.604

[38] Kachergis G, Cox GE, Shiffrin RM. The effects of repeated sequential context on recognition memory. In M. Knauff, M. Pauen, N. Sebanz, & I. Wachsmuth (Eds.), Proceedings of the 35th Annual Conference of the Cognitive Science Society. 2013 July 31 –Aug 3; Berlin, Germany.

[39] Barr DJ, Levy R, Scheepers C, Tily HJ. Random effects structure for confirmatory hypothesis testing: Keep it maximal. Journal of Memory and Language. 2013;68(3):255–78. 10.1016/j.jml.2012.11.001 . PMC3881361 24403724

[40] Bates DM, M.; Bolker, B.; Walker, S. lme4: Linear mixed-effects models using Eigen and S4. In: 1.1–7 Rpv, editor. 2014.

[41] Baayen RH. Analyzing Linguistic Data A Practical Introduction to Statistics Using R, Cambridge University Press 2008

[42] Ferreira VS, Slevc LR, Rogers ES. How do speakers avoid ambiguous linguistic expressions? Cognition. 2005;96(3):263–84. 10.1016/j.cognition.2004.09.002 . 15996561

[43] Alario FX, Segui J, Ferrand L. Semantic and associative priming in picture naming. Q J Exp Psychol-A. 2000;53(3):741–64. 10.1080/713755907 . 10994228

[44] Abdel Rahman R, Melinger A. When bees hamper the production of honey: Lexical interference from associates in speech production. Journal of Experimental Psychology: Learning, Memory, and Cognition. 2007;33(3):604–14. 10.1037/0278-7393.33.3.604 . 17470008

[45] Abdel Rahman R, Melinger A. Semantic context effects in language production: A swinging lexical network proposal and a review. Lang Cognitive Proc. 2009;24(5):713–34. 10.1080/01690960802597250

[46] Griffin ZM. Frequency of meaning use for ambiguous and unambiguous words. Behav Res Meth Ins C. 1999;31(3):520–30. ISI:000082682700015. 10502874 10.3758/bf03200731 PMC5845774

[47] Harm MW, Seidenberg MS. Computing the meanings of words in reading: Cooperative division of labor between visual and phonological processes. Psychological Review. 2004;111(3):662–720. 10.1037/0033-295x.111.3.662 . 15250780

[48] de Zubicaray, McMahon KL. Auditory context effects in picture naming investigated with event-related fMRI. Cognitive Affective & Behavioral Neuroscience. 2009;9(3):260–9. 10.3758/Cabn.9.3.260 . 19679762

[49] Salthouse TA, Hedden T. Interpreting reaction time measures in between-group comparisons. J Clin Exp Neuropsyc. 2002;24(7):858–72. 10.1076/jcen.24.7.858.8392 . 12647765

[50] Faust ME, Balota DA, Spieler DH, Ferraro FR. Individual differences in information-processing rate and amount: Implications for group differences in response latency. Psychological Bulletin. 1999;125(6):777–99. . 10589302 10.1037/0033-2909.125.6.777

[51] Strick M, Holland RW, van Baaren RB, van Knippenberg A. Finding Comfort in a Joke: Consolatory Effects of Humor Through Cognitive Distraction. Emotion. 2009;9(4):574–8. 10.1037/A0015951 . 19653782

[52] Bolte A, Goschke T, Kuhl J. Emotion and intuition: Effects of positive and negative mood on implicit judgments of semantic coherence. Psychological Science. 2003;14(5):416–21. 10.1111/1467-9280.01456 . 12930470

[53] Kiefer M, Schuch S, Schenck W, Fiedler K. Mood states modulate activity in semantic brain areas during emotional word encoding. Cerebral Cortex. 2007;17(7):1516–30. Epub 2006/08/24. 10.1093/cercor/bhl062 . 16926240

[54] Chao LL, Weisberg J, Martin A. Experience-dependent modulation of category-related cortical activity. Cerebral Cortex. 2002;12(5):545–51. Epub 2002/04/16. . 11950772 10.1093/cercor/12.5.545

[55] Storbeck J, Clore GL. The affective regulation of cognitive priming. Emotion. 2008;8(2):208–15. 10.1037/1528-3542.8.2.208 . 18410195 PMC2376275

