# Correction: Listening to Puns Elicits the Co-Activation of Alternative Homophone Meanings during Language Production

**DOI:** 10.1371/journal.pone.0175831

**Published:** 2017-04-10

**Authors:** The PLOS ONE Staff

The third author’s name is cited incorrectly. The publisher apologizes for the error.

The correct citation is: Rose SB, Spalek K, Abdel Rahman R (2015) Listening to Puns Elicits the Co-Activation of Alternative Homophone Meanings during Language Production. PLoS ONE 10(6): e0130853. doi:10.1371/journal.pone.0130853
