# Supplementary material for: Listening to Puns Elicits the Co-Activation of Alternative Homophone Meanings during Language Production
Source: PLoS One. 2015 Jun 26;10(6):e0130853. doi: 10.1371/journal.pone.0130853 (PMC4482729; doi:10.1371/journal.pone.0130853)
Supplement: S1 Table — (DOCX) [file pone.0130853.s001.docx]

S1 Table: Used stimuli material

| **Target** | **Distractor** | | **Primes** | | **Distractor for primes** |
| --- | --- | --- | --- | --- | --- |
| (depicted meaning  vs. non-depicted meaning) | **related** | **unrelated** | **related** | **unrelated** | **unrelated** |
| Flügel (wing  vs. piano) | Cello  (cello) | Kerker  (dungeon) | Gitarre  (guitar) | Pumpe  (water pump) | Sauna  (sauna) |
| Erde (earth  vs. soil) | Sand  (sand) | Cello  (cello) | Torf  (peat) | Feuerwehrmann  (fireman) | Klingel  (bell) |
| Bulle (bull  vs. cop) | Soldat  (soldier) | Tastatur  (keyboard) | Feuerwehrmann  (fireman) | Pudding  (flummery) | Wurzel  (root) |
| Nagel (iron nail  vs. fingernail) | Hand  (hand) | Soldat  (soldier) | Fuß  (foot) | Gitarre  (guitar) | Prisma  (prism) |
| Abzug (trigger  vs. outlet) | Ventilator  (fan/ventilator) | Stuhl  (chair) | Heizlüfter  (fan heater) | Porsche  (Porsche) | Reck  (high bar) |
| Bank (bank  vs. bench) | Stuhl  (chair) | Diskette  (floppy disk) | Hocker  (stool) | Torf  (peat) | Edding  (permanent marker) |
| Schloss (lock  vs. castle) | Palast  (palace) | Bein  (leg) | Laube  (bower) | Heizlüfter  (fan heater) | Delphin  (dolphin) |
| Hahn (cock  vs. water tap) | Sprenger  (sprinkler) | Reis  (rice) | Pumpe  (water pump) | Laube  (bower) | Gürtel  (belt) |
| Mini (mini-skirt  vs. car) | Smart  (Smart) | Ventilator  (fan/ventilator) | Porsche  (Porsche) | Wertpapier  (commercial paper) | Schaf  (sheep) |
| Maus (mouse  vs. computer mouse) | Tastatur  (keyboard) | Pappe  (board paper) | Joystick  (joystick) | Steinbruch  (quarry) | Ampel  (traffic light) |
| Zelle (cell  vs. holding cell) | Kerker  (dungeon) | Sprenger  (sprinkler) | Vogelkäfig  (birdcage) | Brokkoli  (broccoli) | Diadem  (tiara) |
| Linse (lens  vs. lentil) | Reis  (rice) | Palast  (palace) | Brokkoli  (broccoli) | Hocker  (stool) | Schuh  (shoe) |
| Barren (parallel bars  vs. ingot) | Münze  (coin) | Sand  (sand) | Diamant  (diamond) | Heft  (notebook) | Pissoir  (urinal) |
| Gericht (court  vs. dish) | Imbiss  (snack) | Hand  (hand) | Pudding  (flummery) | Schnabel  (beak) | Zange  (pliers) |
| Mine (reservoir  vs. mine) | Tunnel  (tube) | Imbiss  (snack) | Steinbruch  (quarry) | Vogelkäfig  (birdcage) | Löwenzahn  (dandelion) |
| Hacke (mattock  vs. heel) | Bein  (leg) | Scheck  (check) | Knie  (knee) | Diamant  (diamond) | Würfel  (dice) |
| Kohle (coal  vs. cash) | Scheck  (check) | Geweih  (antlers) | Wertpapier  (commercial paper) | Fuß  (foot) | Axt  (axe) |
| Blatt (leaf  vs. sheet) | Pappe  (paperboard) | Münze  (coin) | Heft  (notebook) | Stick  (pen drive) | Falter  (moth) |
| Horn (bugle  vs. animal horn) | Geweih  (antlers) | Smart  (Smart) | Schnabel  (beak) | Joystick  (joystick) | Unterhemd  (undershirt) |
| Chip (crisps  vs. chip) | Diskette  (floppy disk) | Tunnel  (tube) | Stick  (pen drive) | Knie  (knee) | Rechen  (rake) |
| Pickel (pickaxe  vs. pimple) | Ekzem  (eczema) | Athlet  (athlete) | Herpes  (herpes) | Wein  (wine) | Tulpe  (tulip) |
| Iris (flower  vs. iris) | Wimper  (eyelash) | Kratzer  (scratch) | Zunge  (tongue) | Pfanne  (frying pan) | Schal  (scarf) |
| Fliege (bowtie  vs. fly) | Moskito  (mosquito) | Wimper  (eyelash) | Assel  (woodlouse) | Grapefruit  (grapefruit) | Spaten  (spade) |
| Brille (toilet seat  vs. glasses) | Monokel  (monocle) | Schulter  (shoulder) | Fernglas  (binoculars) | Jockey  (jockey) | Lebkuchen  (gingerbread) |
| Melone (bowler  vs. melon) | Kürbis  (pumpkin) | Besteck  (cutlery) | Grapefruit  (grapefruit) | Whiteboard  (whiteboard) | Rohr  (pipe) |
| Boxer (dog  vs. sportsman) | Athlet  (athlete) | Schere  (scissors) | Jockey  (jockey) | Pfirsich  (peach) | Holz  (wood) |
| Käfer (beetle  vs. bug) | Spinne  (spider) | Oma  (grandma) | Hummel  (bumblebee) | Herpes  (herpes) | Mond  (moon) |
| Tafel (table  vs. blackboard) | Flipchart  (flip chart) | Spinne  (spider) | Whiteboard  (whiteboard) | Akkordeon  (accordion) | Panzer  (tank) |
| Pfeife (pipe  vs. fife) | Tuba  (Saxofon) | Selter  (soda) | Akkordeon  (accordion) | Baby  (baby) | Kamm  (comb) |
| Birne (bulb  vs. pear) | Apfel  (apple) | Ekzem  (eczema) | Pfirsich  (peach) | Kranz  (wreath) | Hebel  (lever) |
| Schimmel (white horse  vs. mildew) | Keim  (germ) | Tuba  (Saxofon) | Champignon  (champignon) | Haare  (hair) | Brief  (letter) |
| Bienenstich (bee sting cake  vs. sting of a bee) | Kratzer  (scratch) | Gesteck  (flower arrangement) | Narbe  (scar) | Fernglas  (binoculars) | Lineal  (ruler) |
| Brause (douche  vs. limo) | Selter  (soda) | Pelz  (fur) | Wein  (wine) | Assel  (woodlouse) | Bildschirm  (display) |
| Nadel (fir needle  vs. needle) | Schere  (scissors) | Apfel  (apple) | Pinzette  (tweezers) | Champignon  (champignon) | Handschuh  (glove) |
| Strauß (ostrich  vs. bouquet) | Gesteck  (flower arrangement) | Zimtstern  (cinnamon star) | Kranz  (wreath) | Narbe  (scar) | Regal  (shelf) |
| Becken (cymbal  vs. pelvis) | Schulter  (shoulder) | Kürbis  (pumkin) | Wirbelsäule  (backbone) | Pinzette  (tweezers) | Anorak  (anorak) |
| Stollen (tunnel  vs. Christmas stollen) | Zimtstern  (cinnamon star) | Keim  (germ) | Torte  (cake) | Wirbelsäule  (backbone) | Hai  (shark) |
| Feder (spring  vs. feather) | Pelz  (fur) | Moskito  (mosquito) | Haare  (hair) | Torte  (cake) | Sattel  (saddle) |
| Mutter (screw nut  vs. mother) | Oma  (grandma) | Flipchart  (flip chart) | Baby  (baby) | Hummel  (bumblebee) | Vitrine  (showcase) |
| Geschirr (horse gear  vs. tableware) | Besteck  (cutlery) | Monokel  (monocle) | Pfanne  (frying pan) | Zunge  (tongue) | Dill  (dill) |
| Gabel (fork  vs. yoke) | Pedale  (pedal) | Bauch  (belly) | Kugellager  (axle bearing) | Arena  (arena) | Vorhang  (curtain) |
| Speiche (spoke  vs. radius - bone) | Ellbogen  (elbow) | Party  (party) | Brust  (chest) | Tuch  (neckerchief) | Sessel  (armchair) |
| Brücke (bridge  vs. pontic) | Implantat  (implant) | Schachtel  (box) | Zahnschutz  (gum shield) | Flokati  (flokati rug) | Pfifferling  (chanterelle) |
| Ring (ring  vs. boxing ring) | Manege  (manege) | Pflock  (stake) | Arena  (arena) | Wasserfall  (waterfall) | Geier  (vulture) |
| Bahn (train  vs. path) | Piste  (racing track) | Matte  (mat) | Slalom  (slalom) | Dübel  (wall plug) | Kolibri  (hummingbird) |
| Kreuz (cross  vs. lower back) | Bauch  (belly) | Tablett  (tray) | Hüfte  (hip) | Lobby  (lobby) | Rosmarin  (rosemary) |
| Ball (toy  vs. prom) | Party  (party) | Klinke  (handle) | Oktoberfest  (Oktoberfest) | Scharnier  (butt hinge) | Heizung  (heating) |
| Strudel (strudel  vs. swirl) | Brandung  (breakwater) | Pedale  (pedal) | Wasserfall  (waterfall) | Kugellager  (axle bearing) | Schmetterling  (butterfly) |
| Schalter (switch  vs. counter) | Kasse  (till) | Gestrick  (knitted fabric) | Tresen  (counter) | Blech  (griddle) | Papagei  (parrot) |
| Kapelle (chapel  vs. band) | Band  (band) | Floh  (flea) | Orchester  (orchestra) | Zahnschutz  (gumshield) | Stiefel  (boot) |
| Pflaster (patch  vs. pavement) | Asphalt  (tarmac) | Manege  (manege) | Bordstein  (curb) | Hüfte  (hip) | Korsett  (bodice) |
| Platte (music record  vs. platter) | Tablett  (tray) | Kammer  (chamber) | Blech  (griddle) | Slalom  (slalom) | Hemd  (shirt) |
| Diele (floorboard  vs. hallway) | Kammer  (chamber) | Implantat  (implant) | Lobby  (lobby) | Orchester  (orchestra) | Pavian  (baboon) |
| Läufer (runner  vs. carpet) | Matte  (mat) | Gehörgang  (ear canal) | Flokati  (flokati rug) | Bordstein  (curb) | Cocktail  (cocktail) |
| Angel (fishing rod  vs. garnet hinge) | Klinke  (handle) | Piste  (racing track) | Scharnier  (butt hinge) | Brust  (chest) | Couch  (couch) |
| Muschel (clam  vs. ear conch) | Gehörgang  (ear canal) | Band  (band) | Nase  (nose) | Tresor  (safe) | Kuchen  (cake) |
| Kassette (tape  vs. cash box) | Schachtel  (box) | Brandung  (breakwater) | Tresor  (safe) | Wurm  (worm) | Ofen  (stove) |
| Futter (feed  vs. lining) | Gestrick  (knitted fabric) | Ellbogen  (elbow) | Tuch  (neckerchief) | Nase  (nose) | Bleistift  (pencil) |
| Bremse (brake  vs. gadfly) | Floh  (flea) | Asphalt  (tarmac) | Wurm  (worm) | Tresen  (counter) | Kakao  (cacao) |
| Hering (herring  vs. tent peg) | Pflock  (stake) | Kasse  (till) | Dübel  (wall plug) | Oktoberfest  (Oktoberfest) | Sofa  (sofa) |
