# Supplementary material for: Listening to Puns Elicits the Co-Activation of Alternative Homophone Meanings during Language Production
Source: PLoS One. 2015 Jun 26;10(6):e0130853. doi: 10.1371/journal.pone.0130853 (PMC4482729; doi:10.1371/journal.pone.0130853)
Supplement: S2 Table — (DOCX) [file pone.0130853.s002.docx]

S2 Table: Used jokes and puns

| **Jokes** | **Puns**  *(*********no humorous translation in English possible.)* |
| --- | --- |
| Als ich noch jünger war, hasste ich es auf Hochzeiten zu gehen. Meine beiden Großmütter und alle möglichen Tanten drängten sich um mich herum und kicherten: Du bist der nächste, du bist der nächste. Sie haben erst mit dem ganzen Mist aufgehört als ich anfing auf Beerdigungen das Selbe zu machen.  *When I was younger I hated to go to weddings. My two grandmas and all of my aunts gathered around me and giggled: You’re next, you’re next. They only stopped that rubbish (bullshit) when I started doing the same to them at funerals.* | "Sie sind aber schnell von Ihrer Runde zurück!", sagt der Trainer zur Golferin. "Ich bin von einer Biene gestochen worden", antwortet sie. "Wo denn?", fragt er. "Zwischen dem ersten und dem zweiten Loch!".  Darauf der Trainer: "Ich habe Ihnen schon immer gesagt, dass Ihr Stand zu breit ist!"  *“You’re back early from your round!”, says the coach to the female golfer. “I got stung by a bee”, she replies. “Where?” asks the coach. „Between the first and second hole. „The coach replies: “I always said that your stand was too wide.”* |
| Die Mutter klärt ihre Tochter auf: „Dort wo der Samen reingeht, kommt auch das Baby raus.“ Entsetzt greift sich die Tochter an den Hals und sagt: „Meine Güte, das wird eng.“  *A mother explains to her daughter: “The baby will come out where the sperm goes in”. Frightened the daughter touches her throat: “Gosh that will be tight.”* | Treffen sich zwei Streichhölzer. Sagt das Eine zum Anderen: "Hast du mal 'nen Euro?“. Darauf das Andere: "Nee. Ich bin vollkommen abgebrannt!"  *Two matches meet. One says to the other: “Do you have a dollar? „The other replies: “Sorry but I’m stone-broke.”** |
| Wer waren die ersten drei Politiker? Die Heiligen Drei Könige. Sie legten die Arbeit nieder, zogen sich schöne Gewänder an und gingen ständig auf Reisen.  *Who were the first three politicians? The three Magi. They stopped working, put on some nice robes and always hit the road.* | Ein Brett begegnet einem Stein und fragt: „Was bist du denn für einer?“ Antwortet der Stein: „Ich bin ein Stein/Einstein!“ Entgegnet das Brett: „Wenn du Einstein bist, bin ich Brett Pitt/Brad Pitt!“  *A board meets a stone and asks: “What are you?” The stone answers: “I’m a stone!” The board replies: “If you are a stone, then I’m Brad Pitt…”** |
| Was macht eine Blondine, die vor einem Cerankochfeld steht? Sie wartet auf grün.  *What’s a blond doing in front of a ceramic glass cooktop? Waiting for the light to turn green.* | „Was ist eine Blumenehe?“  „Sie verwelkt, er verduftet!“  *“What’s a flower-marriage?”*  *“She fades, he vamooses.”* ******* |
| Zwei Politiker unterhalten sich. Sagt der eine: „Herr Kollege, was sagten sie doch neulich in ihrer großartigen Rede über die Jugendarbeitslosigkeit?“ „Nichts.“ „Das ist mir schon klar. Ich wollte nur wissen wie sie es formuliert haben.“  *Two politicians have a conversation. One says: “What did you say last time in your brilliant speech about youth unemployment?”; “Nothing.”; “Well, I know that, but I was wondering how you expressed it.”* | 500 Polizisten haben vor dem Berliner Reichstag einen Lieferwagen umstellt. Auf der Windschutzscheibe klebt ein Zettel: „Komm gleich wieder! Bin Laden.“    *500 policemen surround a van in front of the Berlin Reichstag.*  *On the front windshield there’s a note: “Be back soon! Bin Laden (I’m in market).” ** |
| Die Mutter schimpft mit ihrer 15 jährigen Tochter. „Mit 15 Jahren schon bei einem Freund übernachten und dann auch noch den dreißigsten Geburtstag der eigenen Mutter vergessen.“  *A mother tells her 15 years old daughter off.*  *“Not only that you are just 15 years old and stay all night at your boyfriend’s house but you also forgot your own mother’s 30^th^ birthday!”* | Was ist der Unterschied zwischen einem Politiker und einem Telefonhörer?  Den Telefonhörer kann man aufhängen, wenn man sich verwählt hat!  *What the difference between a politician and a telephone receiver? You can actually hang up the receiver if you dialed the wrong number.* |
| Gehen zwei Zahnstocher spät abends am Straßenrand dahin. Plötzlich werden sie von einem Igel überholt. Meint der eine Zahnstocher zum anderen: „So ein Mist, der Bus wäre ja auch noch gefahren.“  *Two toothpicks walk along the roadside at night. Suddenly, a hedgehog passes by. One toothpick says to the other: “Damn it, we could have taken the bus.”* | Eine einsame Katze geht in ein Restaurant und verlangt einen großen Wodka. Der Wirt erklärt, dass Katzen bei ihm keinen Wodka bekämen. „Schade!“, sagt die Katze traurig, „Ich habe gehört, davon bekommt man einen Kater!“  *A lonesome cat walks into a restaurant and asks for a double shot of vodka. The bartender explains that cats don’t receive alcohol from him. „Oh, that’s too bad”, replies the cat sadly, “I heard that you get a hangover from it.” ** |
| Klettert eine junge Schildkröte einen Baum hoch und springt von einem langen Ast herunter. Sie kommt unbeschädigt auf, klettert nochmal hoch und springt wieder. Neugierig beobachtet ein Taubenpaar die kleine Schildkröte, wie sie wieder und wieder springt. Sagt die eine Taube zur anderen: „Du Schatz, wir sollten ihr endlich sagen, dass wir sie adoptiert haben“.  *A young turtle climbs up a tree and jumps off a long branch. She lands safely on the floor, climbs up the tree once again and jumps again. Out of curiosity, two doves observe the young turtle jumping over and over again. One dove says to the other: “Honey, we should finally tell her that she is adopted.”* | Ruft Häschen in einer Molkerei an.  „Hattu Milch?“  „Ja, selbstverständlich!“  „Hattu auch Fettarme?“  „Natürlich!“  „Muttu langärmlige Blusen tragen!“  *Bunny calls the dairy.*  *“Do you have milk?” “Well, of course!”*  *“Do you have skimmed milk?”*  *“Of course!”*  *“Well, then you should wear long-sleeves.”* ******* |
| Treffen sich zwei Schnecken. Die eine hat ein blaues Auge. Fragt die andere: „Warum hast du denn ein blaues Auge?“ Sagt die eine: „Ja, als ich gestern so durch den Wald gerast bin, schießt auf einmal ein Pilz aus dem Boden.“  *Two snails meet. The one snail has a black eye. The other snail asks him: “What happened, why do you have a black eye?”; The other one replies: “Well, yesterday, I was running really fast through the woods when suddenly a mushroom popped up.* | „Warum gehen Ameisen nicht in die Kirche?“  „Weil sie Insekten/in Sekten sind!“  *“Why don’t ants go to church?”*  *“Because they are in sects.”* |
| Im alten Jerusalem will eine wildgewordene Masse von Menschen gerade eine Ehebrecherin steinigen. Jesus geht dazwischen und hält eine Predigt, die damit endet: „Wer von euch ohne Sünde ist, der werfe den ersten Stein.“ Da fliegt ihm mit voller Wucht ein Stein ins Genick. Jesus dreht sich um und brüllt: „Oh Mutter, du nervst langsam.“  *In ancient Jerusalem, a furious crowd wants to stone an adulteress. Jesus intermediates and preaches a sermon: “Let any one of you who is without sin be the first to throw a stone. “ Suddenly a stone hits him hard right on the neck. Jesus turns around and yells: ”Mother! I’m so fed up with you!”* | „Florian“, sagt der Lehrer, „Nenne mir einen griechischen Dichter!“  „Achilles!“, antwortet Florian.  „Achilles war doch kein Dichter!“, tadelt der Lehrer.  „Aber er ist doch durch seine Verse/Ferse berühmt geworden!“  *“Florian”, says the teacher, “Name a Greek poet!”*  *“Achilles!” answers Florian.*  *“Achilles was not a poet!” the teacher retorts.*  *“But he gained fame through his heel/verse.” ** |
| Stehen zwei Kühe im Stall. Sagt die eine: „Muh.“ Sagt die andere: “Mist, das wollte ich auch gerade sagen.“  *Two cows are in the barn. One says: “Moo.” The other says: “Damn it, that’s exactly what I wanted to say.”* | „Was macht ein schwuler Storch?“  „Er fliegt zu seinem Horst!“  *“What does a gay stork do?”*  *“He flies to his Horst (aerie).”* ******* |
| „Jedes Mal wenn sie mich so anlachen Fräulein Franke, würde ich sie am liebsten fragen, ob sie nicht einmal zu mir kommen möchten.“ Sie sind vielleicht ein Schmeichler.“ „Nein, Zahnarzt.“  *Every time when you smile at me like this Miss Franke, I want to ask you whether you want to come visit me.” “You are such a charmer!” “No, I’m a dentist.”* | Ein Ehepaar sitzt auf dem Balkon.  Sie sieht verträumt in den Sonnenuntergang und säuselt romantisch: „Hör mal, Schatz! Die Grillen/grillen!“ Er schaut auf: „Ich riech nichts!“  *A couple sits on a balcony.*  *In a dreamy mood, the woman looks into the sunset and romantically murmurs: “Listen honey, the grilles.”*  *He looks up: “I don’t smell anything!” ** |
| Tim sieht niedergeschlagen aus. „Was hast du denn?“ fragt ihn sein bester Freund.  „Meine Freundin unterstellt mir, dass ich zu neugierig bin.“ „Wie kommst du denn darauf?“  „Na, ich habe es doch schwarz auf weiß in ihrem Tagebuch gelesen.“  *Tim looks devastated.  “What is going on?” asks his best friend. “My girlfriend thinks I’m too curious.” “How did you come up with this idea?” “Well, I read it in black and white in her diary.”* | Der Begriff „Labormaus“ wird völlig abgeschafft. Das heißt „medizinisch-technische Assistentin“!  *The term laboratory mouse is no longer used. We refer to them as medical technical assistant.”* |
| Zwei Blumen wachsen nebeneinander.  „Liebling, ich liebe dich.“  „Ich liebe dich auch. Ich will dich.“  „Ich will dich auch. Wo sind diese verdammten Bienen?“  *Two flowers grow next to each other.  “Sweetheart, I love you.” “I love you too! I want you!” “I want you too! Where the hell are the damned bees?”* | „Wie nennt man einen intelligenten Toilettenbenutzer?“  „Klugscheißer!“  *“What do you call an intelligent toilet user?”*  *“A smartass.”* |
| Zwei Australier unterhalten sich.  Sagt der eine: „Stell dir vor, mein Nachbar hat einen Schädelbasisbruch.“ „Wie ist denn das passiert?“, fragt der andere. „Nun er hat sich einen neuen Bumerang gekauft und den alten weggeworfen.“  *Two Australians have a conversation.  One says: “Imagine, my neighbor has a basal skull fracture.” - “How did that happen?”, asks the other.*  *“Well, he bought a new boomerang and threw the old one away.”* | Treffen sich zwei Ziegen. Sagt die Eine: „Gehst du heut‘ Abend mit aufs Fest?“Darauf die Andere: „Nee. Ich hab‘ kein‘ Bock!“  *Two goats meet. One says: “Will you come to the party tonight?”The other replies: “No, couldn’t be buggered.”** |
| Meint die eine Mutter zur anderen: „Mein Sohn meditiert neuerdings den ganzen Tag.“  Ich weiß zwar nicht genau was das ist, aber sicher besser als rumsitzen und nichts tun.“  *One mother says to another: “Recently, my son started meditating all day long. Even though I don’t know exactly what that is, it’s surely better than sitting around and doing nothing at all.”* | „Na, was habt ihr denn in der Schule heute Schönes gemacht?“, fragt die Mutter ihre Tochter. „Och, wir haben männliche Prostituierte gemalt!“  „Ihr habt *was* gemalt?“ - „Na, Strichmännchen!“  *“What did you do in school today?” the mother asks her daughter. “We painted male prostitutes.” “You did what?”*  *“We painted stickmen!”******** |
| Kurz vor der Geburt:  Die Hebamme fragt die werdende Mutter: „Und, wünschen sie, das der Vater bei der Geburt dabei ist?“  Antwortet die werdende Mutter: „Um Gotteswillen nein, mein Mann kann ihn absolut nicht leiden.“  *Shortly before childbirth:*  *The midwife asks the mother-to-be: “Do you want the father to attend the birth?” The mother-to-be replies: “For heaven’s sake no, my husband can’t stand him.”* | „Was hat ein Engel, der in den Mist gefallen ist?“  „Kotflügel!“  *“What does an angel that fell into a pile of dung possess?” - “Mud wings!”** |
| Kommt eine Amsterdamer Prostituierte in ein Bahnabteil. Fragt sie höflich ein Reisender: „Möchten sie am Fenster sitzen?“  „Nein danke, ich habe heute frei.“  *A prostitute from Amsterdam enters a train compartment. “Do you want to take the window seat?”, a polite traveler asks. “No thank you, it’s my day off.”* | Inschrift auf dem Grabstein eines Lehrers: „Ein treues Herz und zwei nimmermüde Hände haben aufgehört zu schlagen!“  *Inscription on a teacher’s gravestone: “A faithful heart and two tireless hands stopped beating.”* |
| Was hört man wenn man sich einen Döner ans Ohr hält? Das Schweigen der Lämmer.  *What do you hear when you listen to a doner kebab? The silence of the lambs.* | Zwei Kannibalen essen einen Clown.  Sagt der Eine zum Anderen: „Hm. Schmeckt irgendwie komisch“.  *Two cannibals are eating a clown.*  *One says to the other: “It tastes kind of funny.”* |
| „Herr Doktor, wenn ich auf mein Knie drücke tut es weh und wenn ich auf mein Hinterteil drücke ebenfalls.“  „Mmh ja, Ihr Finger ist gebrochen.“  *“Doctor, doctor, when I touch my knee it hurts and when I press on my butt it hurts as well.”*  *“Hmm, yes, your finger is broken”.* | Inschrift auf dem Grabstein eines Spanners: „Jetzt ist er weg vom Fenster!“  *Inscription on a voyeur’s gravestone:*  *“He is a has-been.” ** |
| „Unfair, wenn ich mit Gott spreche, heißt es Gebet. Aber wenn er mit mir spricht, dann heißt es gleich Psychose.“  *“How unfair: when I talk to God, they call it a prayer. But when he talks to me, they call it a psychosis.”* | „Was ist Viagraoxyd?“  „Ganz klar: Lattenrost!“  *“What is Viagraoxide?”*  *“Clearly: Duckboard.”* ******* |
| Sagt Adam zu Eva: „Liebst du mich?“  Antwort: „Ja, wen denn sonst?“  *Adam to Eve: “Do you love me?”*  *Adam replies: “Yes, who else would I love?”* | „Nimmt Ihre Frau nichts gegen den Hustenreiz?“  „Nein! Es ist der einzige Reiz, den sie noch hat!“  *“Your wife does not take anything against the tussive irritation?”-“No! It’s the only attraction she still has.******** |
| Ein Känguruh hüpft durch den Wald. Immer wieder muss es sich am Bauch kratzen. Dann hat es genug. Es zieht sein Baby aus dem Beutel und sagt: „Wie oft soll ich dir noch sagen du sollst in deinem Zimmer kein Zwieback essen.“  *A kangaroo jumps through the woods. Over and over she itches her tummy. Finally it’s enough. She pulls out her baby from her pouch and says: “How many times do I have to tell you not to eat crackers in your room!.”* | Unterhalten sich zwei Kerzen.  Fragt die eine Kerze die Andere: „Du, sag mal, ist Wasser gefährlich?“  Antwortet die andere: „Da kannst du von ausgehen!“  *Two candles have a conversation.*  *One asks the other: “Is water dangerous?”*  *The other replies: “You can assume so!” ** |
| „Ich soll also mehr Feuer in meine Gedichte legen.“  „Nein, umgekehrt.“  *“So I’m supposed to put more fire into my poems.”*  *“No, the other way around.”* | „Ich habe heute versucht, bei den Weight Watchers anzurufen. Hat aber keiner abgenommen.“  *“I tried to call Weight Watchers today. However, nobody answered.”* ******* |
| Verlassen zwei Taschendiebe ein Restaurant. „Hast du die goldene Uhr des Kellners gesehen?“  „Nein, zeig mal her!“  *Two pickpockets leave a restaurant. “Did you see the waiter’s golden wristwatch?” Says one.*  *“No, show me!”Says the other.* | „Warum trinken die Franzosen Wein, die Russen Wodka und die Deutschen Bier?“  „Damit man die Völker an der Fahne erkennt!“  *“Why do the French drink wine, the Russians vodka and the Germans beer?”*  *“So you can distinguish the nations by their flag.” ** |
