# Supplementary material for: Listening to Puns Elicits the Co-Activation of Alternative Homophone Meanings during Language Production
Source: PLoS One. 2015 Jun 26;10(6):e0130853. doi: 10.1371/journal.pone.0130853 (PMC4482729; doi:10.1371/journal.pone.0130853)
Supplement: S4 Table — (DOCX) [file pone.0130853.s004.docx]

S4 Table: Correlations of fixed effects of the subsequent LMMs separated for the related and unrelated prime condition.
